# Adjuvant re-irradiation vs. no early re-irradiation of resected recurrent glioblastoma: pooled comparative cohort analysis from two tertiary centers

**DOI:** 10.1007/s11060-024-04633-2

**Published:** 2024-03-23

**Authors:** Christoph Straube, Stephanie E. Combs, Denise Bernhardt, Jens Gempt, Bernhard Meyer, Claus Zimmer, Friederike Schmidt-Graf, Peter Vajkoczy, Arne Grün, Felix Ehret, Daniel Zips, David Kaul

**Affiliations:** 1 Department of Radiation Oncology, Klinikum rechts der Isar, Technical University of Munich, Munich, Germany; 2 Department of Radiation Oncology and Radiotherapy, Klinikum Landshut, Landshut, Germany; 3 Department of Neurology, Klinikum rechts der Isar, Technical University of Munich, Munich, Germany; 4 Department of Neurosurgery, Klinikum rechts der Isar, Technical University of Munich, Munich, Germany; 5 Department of Neuroradiology, Klinikum rechts der Isar, Technical University of Munich, Munich, Germany; 6 Department of Neurosurgery, Charité– Universitätsmedizin Berlin, Corporate Member of Freie Universität Berlin and Humboldt-Universität zu Berlin, Berlin, Germany; 7 Department of Radiation Oncology, Charité– Universitätsmedizin Berlin, Corporate Member of Freie Universität Berlin and Humboldt-Universität zu Berlin, Berlin, Germany; 8 Charité– Universitätsmedizin Berlin, Berlin, Germany; 9 Partner Site Berlin, German Cancer Consortium (DKTK), German Cancer Research Center (DKFZ), Heidelberg, Germany; 10 Department of Neurosurgery, University Hamburg-Eppendorf, Hamburg, Germany

**Keywords:** Glioblastoma, Recurrence, Re-irradiation, 2nd surgery, GTR, Gross total resection, Adjuvant radiotherapy, Re-irradiation, Cohort study

## Abstract

**Background:**

The optimal management strategy for recurrent glioblastoma (rGBM) remains uncertain, and the impact of re-irradiation (Re-RT) on overall survival (OS) is still a matter of debate. This study included patients who achieved gross total resection (GTR) after a second surgery after recurrence, following the GlioCave criteria.

**Methods:**

Inclusion criteria include being 18 years or older, having histologically confirmed locally recurrent IDHwt or IDH unknown GBM, achieving MRI-proven GTR after the second surgery, having a Karnofsky performance status of at least 60% after the second surgery, having a minimum interval of 6 months between the first radiotherapy and the second surgery, and a maximum of 8 weeks from second surgery to the start of Re-RT.

**Results:**

A total of 44 patients have met the inclusion criteria. The median OS after the second surgery was 14 months. All patients underwent standard treatment after initial diagnosis, including maximum safe resection, adjuvant radiochemotherapy and adjuvant chemotherapy. Re-RT did not significantly impact OS. However, MGMT promoter methylation status and a longer interval (> 12 months) between treatments were associated with better OS. Multivariate analysis revealed the MGMT status as the only significant predictor of OS.

**Conclusion:**

Factors such as MGMT promoter methylation status and treatment interval play crucial roles in determining patient outcomes after second surgery. Personalized treatment strategies should consider these factors to optimize the management of rGBM. Prospective research is needed to define the value of re-RT after second surgery and to inform decision making in this situation.

**Supplementary Information:**

The online version contains supplementary material available at 10.1007/s11060-024-04633-2.

## Introduction

Currently, there is no established standard of care for recurrent glioblastoma (rGBM) [1]. In this analysis, we evaluated the impact of re-irradiation (Re-RT) on overall survival (OS) in a selected cohort of patients with rGBM who underwent gross total resection (GTR) recurrent disease.

Despite the absence of randomized trials, second surgery is often offered to patients with rGBM, particularly when the tumor is located in a non-eloquent area, has a volume smaller than 50 cm³, and the patient is in good overall condition [2]. Notably, patients who achieve GTR after the second surgery tend to experience improved survival outcomes [3–5].

Our previous findings have indicated that following a second resection achieving GTR, recurrences primarily occur locally, suggesting a potential benefit of early re-irradiation of the resection cavity [6]. Furthermore, patients who underwent GTR have demonstrated better OS after re-RT compared to those who did not achieve GTR, providing support for early RT following GTR [5, 7]. However, it is worth noting that a single-center analysis, which included patients with subtotal resection, has provided initial evidence favoring early re-RT [8].

Contrary to the promising data supporting Re-RT, two recent randomized phase II trials, namely RTOG 1205 and the study by Bergman et al., have challenged the expectations for Re-RT in rGBM. These trials did not observe any improvement in OS attributed to re-RT, although they reported favorable progression free survival (PFS) [9, 10]. These findings contradict several retrospective analyses and prospective single-arm trials that were summarized in a meta-analysis, which supports the use of Re-RT [11].

The concept of early re-irradiation is currently evaluated within the GlioCave-Trial, which is currently recruiting in Germany [12].

## Methods

We conducted a pooled analysis to evaluate the outcomes of patients who underwent GTR for locally rGBM at two tertiary centers: center A and center B. All patients underwent neuro-navigated micro-surgery for recurrent disease, resulting in MRI-proven GTR. The postoperative MRI was performed within 24–72 h after surgery, adhering to current international standards. Noteworthy, the analysis of PFS is beyond the scope of this manuscript.

All patients in our analysis would have met the inclusion criteria for the GlioCave trial (NCT02715297) [12]. These criteria include being 18 years or older, having histologically confirmed locally rGBM, achieving MRI-proven GTR after the second surgery, having a Karnofsky performance status of at least 60% after the second surgery, having a minimum interval of 6 months between the first radiotherapy and the second surgery, and a maximum of 8 weeks from second surgery to the start of Re-RT [12]. Due to the small sample size, patients with proven IDH mutations were not included into this analysis.

We reviewed the patient files to gather information on the initial treatment, salvage treatment, MGMT promoter methylation status, age at re-irradiation, survival time, interval between the first and second surgery, and the treating center. Difference between the re-RT and the non-Re-RT-group were investigated using the ANOVA-Test for numerical variables and the Pearson’s Chi-Square-Test for categorial variables.

If applied, re-irradiation was performed using photons after stereotactic-quality mask fixation. All re-RT procedures were CT- and MRI-planned. The target volume encompassed the resection cavity, including all postoperatively contrast-enhancing regions, with an additional margin of 5 mm to create the clinical target volume (CTV). The planning target volume (PTV) was defined as CTV + 1–2 mm. The dose prescription varied based on the treating physician’s discretion, resulting in a range from 40 Gy/22 to 60 Gy/30. Some patients also received bi-daily treatment with 59.2 Gy/37 BID.

For statistical analysis, overall survival was defined as the time from the second surgery to death. Patients without death event were censored at the last available follow-up. Survival times were estimated using the Kaplan-Meier method, and the log-rank test was employed. A p-value of < 0.05 was considered statistically significant, a p-value of < 0.1 was considered as tendency. To account for potential confounding factors, a multivariate Cox regression analysis was performed to test whether Re-RT has an influence on OS in the context of all factors, that proved significance by the Kaplan-Meier Method. The proportional hazard assumption was tested using the Breslow contact method.

The statistical analysis for this report were conducted using BlueSky-Statistics v10.3.1.

## Results

Out of the total 44 patients included in the analysis, 19 patients were from center A and 25 patients were from center B. Among the patients, 43 had received an initial diagnosis of glioblastoma (GBM), while the initial diagnosis remained unknown for one patient, however, the histological diagnosis at second surgery was IDHwt GBM in this case, too. The MGMT methylation status was available for 36 patients, with 17 cases showing methylation and 19 cases showing non-methylation. The primary treatment for the majority of cases followed the Stupp protocol, consisting of 60 Gy delivered in 30 fractions [12]. The remaining cases received different treatment regimens, including 54 Gy/30, 66 Gy/30, 59.2 Gy/37 BID, or 40.05 Gy/15. The median age at diagnosis was 58 years (range 30 to 77 years), the median age at second surgery was 59 years (range 32 to 78 years). The median interval from end of first RT to second surgery was 13.8 months (range 6.7 to 56.6 months). In all cases, GBM was histologically proven after second surgery. Generally, patients undergoing re-RT were younger, had better performance status and more likely to have a methylated MGMT promotor (Table 1). None of these differences gained significance.

**Table 1:** Patient characteristics

Patient characteristics				
**Re-Rtafter 2nd surgery**	no Re-RT (*N* = 19)	Re-RT (*N* = 25)	Total (*N* = 44)	*p* value
**Age at 1st surgery**				0.113 (1)
- Mean	61.5	55.7	58.2	
- Median (Q1, Q3)	58.8 (54.9, 70.3)	56.0 (48.2, 65.0)	57.4 (51.1, 67.5)	
- Min	39.6	30.9	30.9	
- Max	76.2	77.5	77.5	
**Age at 2nd surgery**				0.128 (1)
- Mean	62.9	57.5	59.8	
- Median (Q1, Q3)	60.5 (55.8, 71.1)	57.5 (50.3, 65.9)	59.0 (52.6, 69.8)	
- Min	44.1	32.2	32.2	
- Max	77.5	78.4	78.4	
**IDH**				
- IDH mt	0 (0.0%)	0 (0.0%)	0 (0.0%)	
- IDH wt	9 (47.3%)	24 (96.0%)	33 (75%)	
- IDH unknown	10 (52.7%)	1 (4.0%)	11 (25%)	
**MGMT**				0.006 (2)
- unmethylated	8 (42.1%)	9 (36.0%)	17 (38.6%)	
- methylated	4 (21.1%)	15 (60.0%)	19 (43.2%)	
- unknown	7 (36.8%)	1 (4.0%)	8 (18.2%)	
**Months from end of last RT to 2nd surgery**				0.201 (1)
- Mean	14.9	18.7	16.7	
- Median (Q1, Q3)	12.2 (8.0, 16.3)	15.4 (10.1, 19.8)	13.8 (9.0, 18.3)	
- Min	6.7	7.7	6.7	
- Max	48.1	56.6	56.6	
**KPS after 2nd surgery**				0.059 (2)
-60%	4 (22.2%)	3 (12.0%)	7 (16.3%)	
-70%	5 (27.8%)	1 (4.0%)	6 (14.0%)	
-80%	6 (33.3%)	7 (28.0%)	13 (30.2%)	
-90%	3 (16.7%)	7 (28.0%)	10 (23.3%)	
-100%	0 (0.0%)	5 (20.0%)	5 (11.6%)	
- n/a	0 (0.0%)	2 (8.0%)	2 (4.7%)	
**Chemotherapy after 2nd surgery**				0.380 (2)
	13 (68.4%)	20 (80.0%)	33 (75.0%)	
Temozolomide	10	17	27	
CCNU		1	1	
CCNU + TMZ		1	1	
CCNU + Bevacizumab	1	1	2	
unkown	2		2	

*Note* (1) Linear Model ANOVA. (2) Pearson’s Chi-squared test

**Fig. 1:**
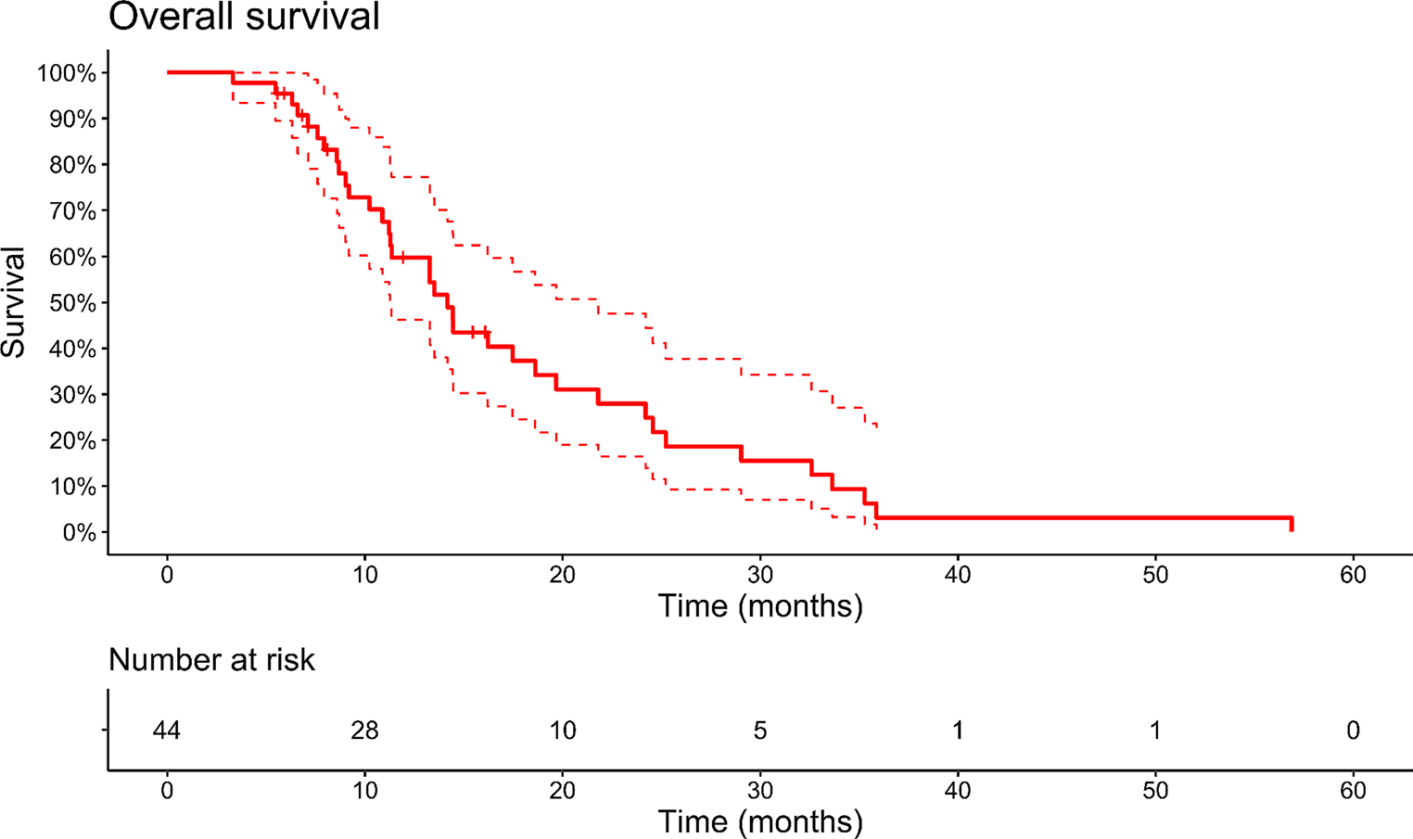
Kaplan-Meier estimates of OS of the entire cohort

The median overall survival (OS) following the second surgery was 14.2 months, with a 95% confidence interval (CI) ranging from 11.3 to 21.8 months (Fig. 1). When analyzing the data separately for center A, the median OS was 14.4 months (95% CI 11.3 months to not defined), while for center B, it was 13.8 months (95% CI 9.2 to 24.6 months), with no statistically significant difference observed (*p* = 0.57).

**Fig. 2:**
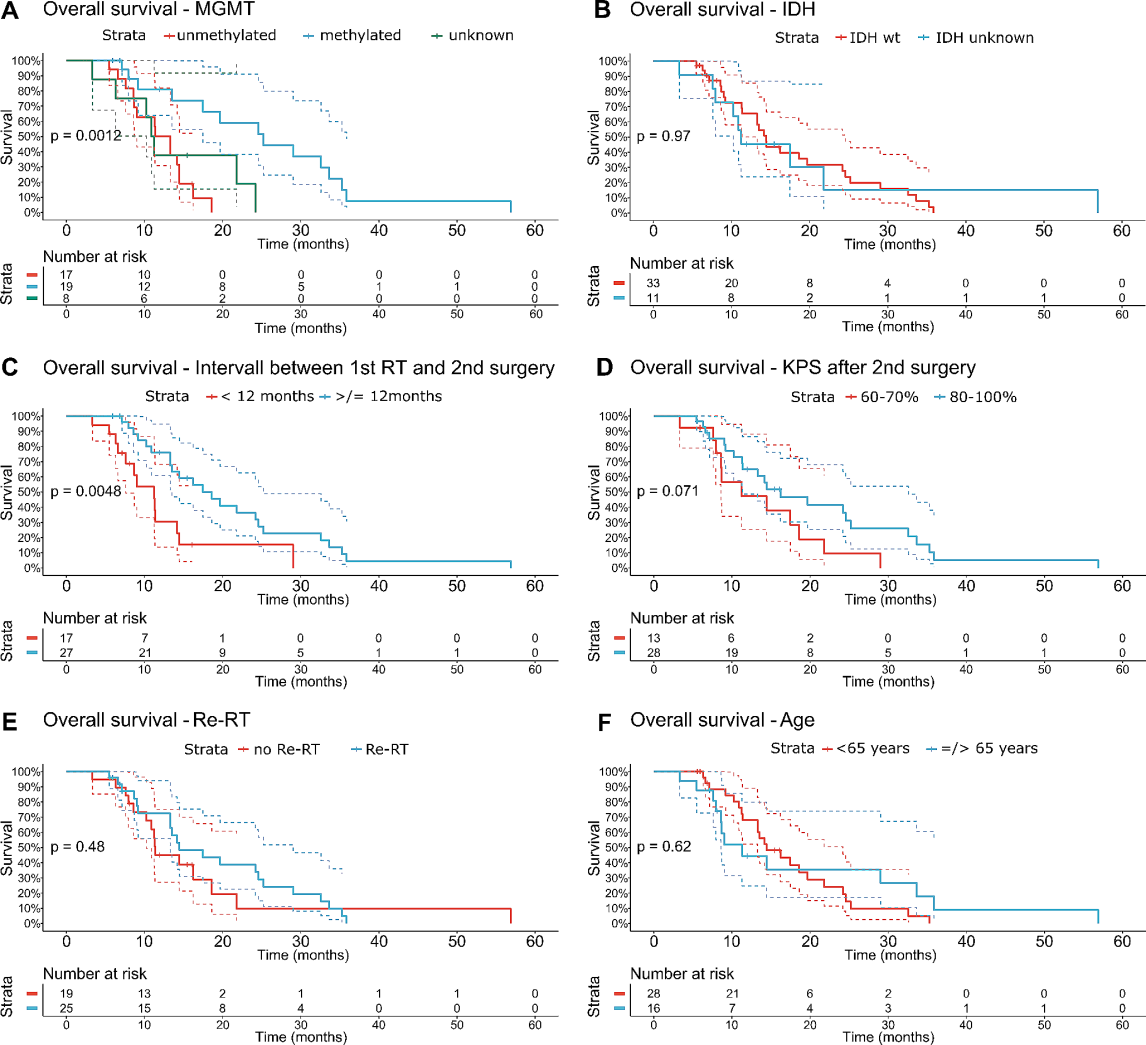
Kaplan-Meier estimates of OS stratified by MGMT promotor methylation status (**A**), by IDH mutation status (**B**), by the interval from the end of first RT to 2nd surgery (**C**), by KPS after second surgery (**D**), by the early adjuvant Re-RT after 2nd surgery, and by the age at 2nd surgery

The MGMT promoter methylation status and the interval between the end of the first radiotherapy (RT) and the second surgery were found to be significant predictors of overall survival (OS). Patients with methylated MGMT promoters had a median OS of 25.2 months (95% CI 17.5 to 35.9 months), whereas patients without methylated promoters had a median OS of 13.3 months (95% CI 8.7 months to NA), demonstrating a statistically significant difference (*p* = 0.001, Fig. 2A).

There was no difference in median OS between patients with wildtype IDH and patients with unknown IDH status (14.4 vs. 11.2 months for IDH_wt_ and IDH_unkown_, respectivly, *p* = 0.97, Fig. 2B).

Similarly, patients with an interval longer than 12 months between progression and subsequent second surgery experienced significantly better OS compared to those with a shorter interval (11.2 months vs. 18.6 months; 95% CI 7.5 months to NA vs. 13.5 to 25.2 months, *p* = 0.0048, Fig. 2C).

The Karnowsky performance status missed significance in this analysis. Patients with a good KPS after second surgery had an improved survival too. A KPS of 60–70% resulted in an OS of 11.2 months vs. 16.2 months in patients with a KPS of 80–100% (*p* = 0.071, Fig. 2D).

Among the cases that received adjuvant re-RT, the median OS was 14.5 months (95% CI 13.3 to 29.0 months), compared to 11.3 months (95% CI 10.2 months to NA) in cases that did not receive Re-RT (Fig. 2E). However, this difference was not statistically significant (*p* = 0.48). Similarly, Re-RT compared to no adjuvant therapy at all did not result in a significant difference, too (median OS 11.3 months, 95% CI 11.2 months– NA, vs. 14.5 months 95% CI 13.3–29.0 months, p 0.55, supplementary figure D). Additionally, the use of chemotherapy following the second surgery, independent of a Re-RT, did not result in a significant difference in OS (*p* = 0.42). An additional exploratory analysis of patients receiving no adjuvant RT did not result in significant differences, too (p 0.83, supplemental figure C). In a further exploratory analysis patients receiving combination therapy had a median survival time of 17.5 months (95% CI 13,3–32.6 months), while those receiving no therapy or monotherapy both had a median survival time of 11.3 months, respectively (95% CI for monotherapy: 8.7–21.8 months; 95% CI for no adjuvant therapy: 11.2– NA months; *p* = 0.53, supplementary Figure A).

The age at re-RT (± 50 years) did not show a significant influence on OS (13.3 vs. 16.2 months, *p* = 0.85, Fig. 2F).

**Fig. 3:**
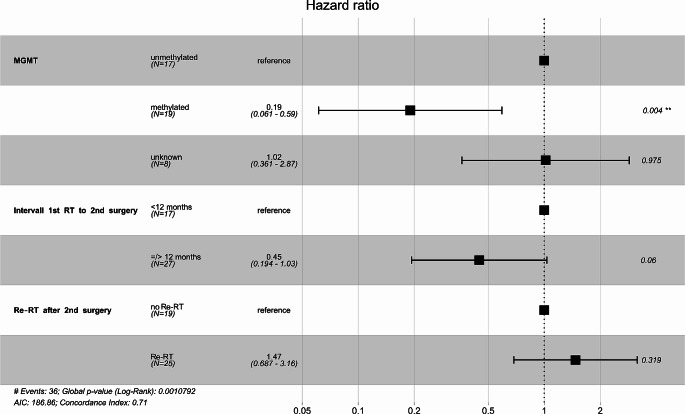
Forest-plot of the multivariate analysis

In an exploratory multivariate analysis, which considered the interval to the second surgery, MGMT promoter methylation status, and Re-RT versus no Re-RT, only a positive MGMT methylation status significantly correlated to a longer OS (Hazard Ratio (HR) 0.19, *p* = 0.004). An interval from 1st surgery to second surgery longer than 12 months resulted in a strong tendency towards a longer OS, slightly missing the criteria for statistical significance (HR 0.45, *p* = 0.06). Adding re-RT did not further improve the quality of the model (HR of 1.47 for Re-RT, 95% CI 0.687 to 3.16, *p* = 0.319, Fig. 3).

## Discussion

In this pooled analysis from two large tertiary centers, we conducted a comparative assessment of patient outcomes undergoing either early adjuvant Re-RT or no adjuvant Re-RT for patients with rGBM after MRI approved GTR. The results did not reveal a significant difference in OS between adjuvant Re-RT and no adjuvant Re-RT. Incorporating re-RT into a multivariate model did not reveal a significant correlation between the use of adjuvant Re-RT and OS. These findings align with recent randomized trials investigating Re-RT for recurrent GBM (rGBM), which have shown an improved PFS but no significant difference in OS [9, 10]. Furthermore, in a retrospective study comparing second surgery with re-RT (repeated radiation therapy) to second surgery without Re-RT, no significant difference in overall survival (OS) was observed, too [8].

Reasons for the lack of OS benefits from adjuvant Re-RT are speculative. Possible explanations could be related to treatment factors, namely inadequate dose or to small target volume. Alternatively, toxic effects could consume the oncologic treatment effect, hence, a PFS benefit would not result in an OS benefit because of lifetime limiting toxicity. One option to enhance the therapeutic ration in Re-RT is the addition of Bevacizumab, a drug known to reduce the incidence of radiation necrosis after Re-RT by 83% [13]. Notably, due to restrictions in re-imbursement in Germany, only one patient received Bevacizumab in the present cohort.

Most of the reported patients received some kind of chemotherapy. However, patients in the Re-RT cohort were slightly more likely to receive chemotherapy as compared to patients in the no Re-RT-cohort. As all patients were deemed to be in a good condition after second surgery, as defined within the inclusion criteria, one would expect an at least equal proportion of patients receiving chemotherapy despite of not receiving adjuvant Re-RT. Hence, the Re-RT group was treated more intensive also in the view of systemic treatments. A recent meta-analysis from Marwah et al. showed that combination therapy increased OS as compared to mono-RT, but it did not increase OS when compared to mono-CTx [13]. Notably, the underlying data are mostly from trials reporting treatment of macroscopic disease. The potential added efficacy of Re-RT might therefore be limited in the context of a former GTR. Consequently, the patient number in our cohort is not large enough to reach significant differences from no adjuvant treatment or mono therapy to Re-RT in combination with chemotherapy.

The data presented in this study offer further insights into prognostic factors for patients with rGBM. Our results highlight the relevance of patient-specific factors, including post-surgery performance status and MGMT methylation status. Furthermore, the time interval between the first RT and the second surgery, which are surrogates for the individual aggressiveness of the disease, has emerged as a crucial contributing factor, resulting in a strong tendency with borderline significance in a multivariate model. These factors, including post-surgery performance status, MGMT methylation status, and the time interval between the first RT and the second surgery, have not only been identified by our research team but also by other researchers [7, 8, 14–17]. Moreover, these factors have been integrated into prognostic scores specifically designed for rGBM [7, 17].

For patients with rGBM, a second surgery is a well-established salvage strategy that should be considered in selected cases. The selection criteria typically include patients with a good performance status, small tumor volume, and a location distant from eloquent areas [18]. Pooled data from multiple large-scale centers in Germany have demonstrated the safety and feasibility of this approach, particularly benefiting patients who have undergone GTR [4]. Additionally, a secondary analysis of the DIRECTOR-Trial, which evaluated the efficacy of two different temozolomide regimens in rGBM, indicated a significant advantage for patients based on the extent of resection. Notably, patients who underwent GTR experienced substantial benefits in this prospective cohort [3]. However, conflicting data exist regarding subtotal resection (STR), with some reports indicating no or even detrimental effects of STR in the DIRECTOR analysis. Conversely, Yang et al. reported a progressively increasing benefit with the relative extent of resection, with even small remnants after the second surgery showing some remaining, albeit diminished, benefit for patients [19]. Furthermore, a meta-analysis of 1906 cases demonstrated a significant improvement in patient outcomes when a second surgery was performed (hazard ratio 0.72, *p* < 0.001) [20]. Therefore, a second surgery for rGBM is– despite a lack of prospective comparative evidence– emerging as a potential treatment approach, particularly in carefully selected patients based on good performance status, small tumor volume, and location distant from eloquent areas [2]. Noteworthy, given the previous selection criteria, our data do not support the use of age as a general exclusion criteria. This also aligns to our previous experience with Re-RT in GBM [21].

The strengths of this article are the homogeneous patient selection in accordance with a published trial protocol as well as the bicentric inclusion of patients, which allows a better generalizability of the data. Limitations are mostly due to retrospective nature of the assessment [22]. Given that positive predictive factors were skewed in favor of patients who received Re-RT, it is unlikely that an effect of Re-RT was missed in this cohort. Exact matching of the cases was not possible due to the low number of patients fulfilling the rigorous inclusion criteria. Additionally, the low patient number reduces the statistical power, a true existing therapeutic effect thus could be overseen. Furthermore, the Re-RT dose regimens were not standardized which introduced some uncertainty, and imaging analysis after Re-RT was not included, hence information on PFS after Re-RT are not available. Additionally, a substantial number of patients had an unknown IDH status. It is known that IDHmt is associated with longer OS after Re-RT [23]. However, since there was no statistical difference in OS between IDHwt and IDH unknown groups, we assume that the IDH unknown cohort had none or very few IDHmt patients which should not influence the overall conclusion of this report. Lastly, analysis of safety was beyond the scope of this article, as comparative data from the control group were not available.

The ongoing GlioCave trial will focus on the impact of Re-RT on PFS. The early results of this randomized trial are eagerly awaited as they might clarify whether Re-RT is safe and can lead to a clinically meaningful improvement in PFS. Importantly, within a palliative setting, a safe treatment that results in a significant improvement in PFS could be an important treatment modality, even in the absence of an OS benefit.

## Conclusion

Our data do not substantiate the hypothesis that early adjuvant Re-RT of the resection cavity can improve the survival outcome of patients after GTR of rGBM. Therefore, adjuvant Re-RT after GTR should be offered within prospective trials and is currently not seen as the standard of care. Besides, factors such as MGMT promoter methylation status, the performance score and treatment interval are shown to play crucial roles in determining patient outcomes after second surgery. Personalized treatment strategies should consider these factors to optimize the management of recurrent glioblastoma.

### Electronic supplementary material

Below is the link to the electronic supplementary material.

Supplementary Material 1

## Data Availability

The dataset supporting the conclusions of this article contains clinical as well as demographic data. Therefore, sharing of the entire dataset online was restricted by the local ethical committee. However, selected data can be requested from the corresponding author.
