# Supplementary figures and images for: Adjuvant re-irradiation vs. no early re-irradiation of resected recurrent glioblastoma: pooled comparative cohort analysis from two tertiary centers
Source: J Neurooncol. 2024 Mar 23;168(1):49–56. doi: 10.1007/s11060-024-04633-2 (PMC11093803; doi:10.1007/s11060-024-04633-2)

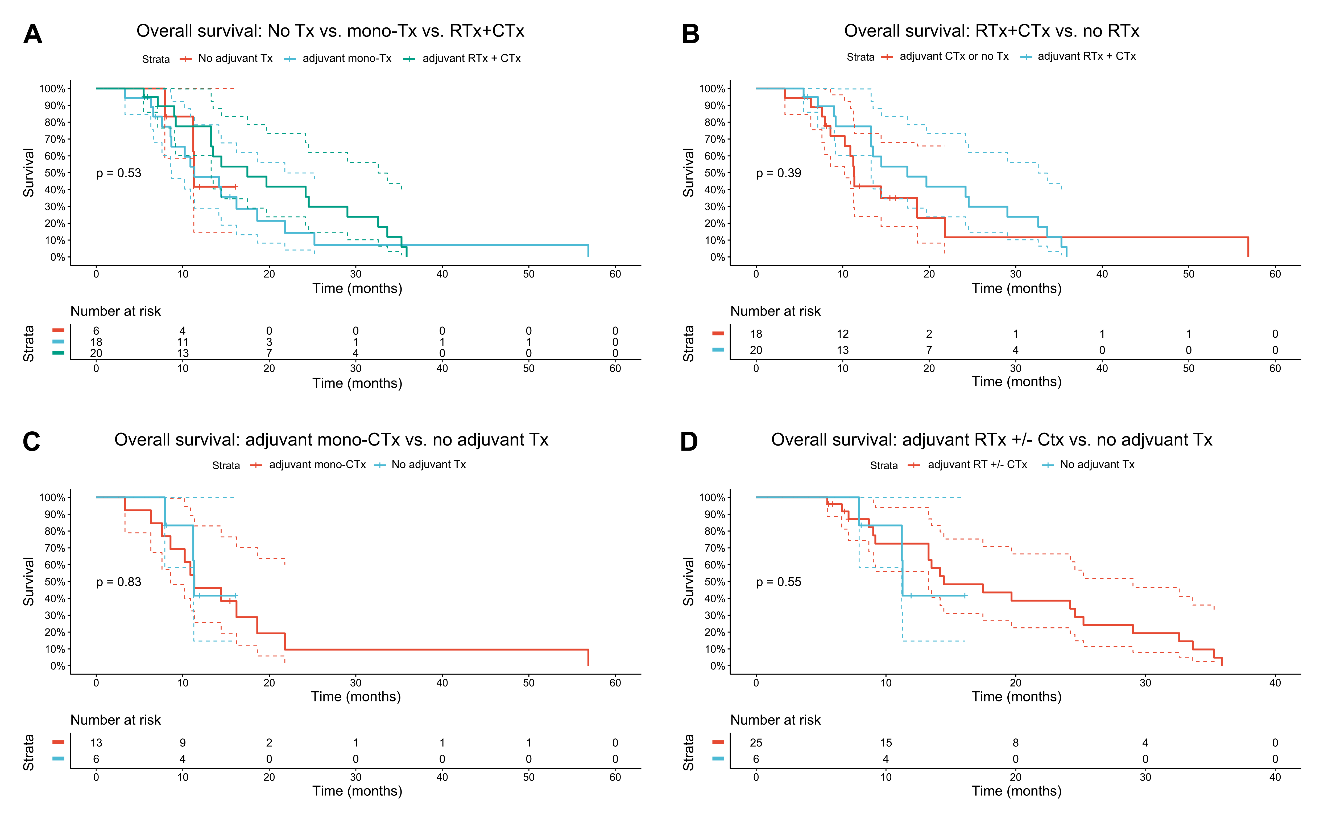

Supplement: Supplementary file 1 — Supplementary Material 1 [file 11060_2024_4633_MOESM1_ESM.docx]
